# Finding evolutionarily conserved cis-regulatory modules with a universal set of motifs

**DOI:** 10.1186/1471-2105-10-82

**Published:** 2009-03-10

**Authors:** Bartek Wilczynski, Norbert Dojer, Mateusz Patelak, Jerzy Tiuryn

**Affiliations:** 1 Institute of Informatics, University of Warsaw, Warsaw, Poland

## Abstract

**Background:**

Finding functional regulatory elements in DNA sequences is a very important problem in computational biology and providing a reliable algorithm for this task would be a major step towards understanding regulatory mechanisms on genome-wide scale. Major obstacles in this respect are that the fact that the amount of non-coding DNA is vast, and that the methods for predicting functional transcription factor binding sites tend to produce results with a high percentage of false positives. This makes the problem of finding regions significantly enriched in binding sites difficult.

**Results:**

We develop a novel method for predicting regulatory regions in DNA sequences, which is designed to exploit the evolutionary conservation of regulatory elements between species without assuming that the order of motifs is preserved across species. We have implemented our method and tested its predictive abilities on various datasets from different organisms.

**Conclusion:**

We show that our approach enables us to find a majority of the known CRMs using only sequence information from different species together with currently publicly available motif data. Also, our method is robust enough to perform well in predicting CRMs, despite differences in tissue specificity and even across species, provided that the evolutionary distances between compared species do not change substantially. The complexity of the proposed algorithm is polynomial, and the observed running times show that it may be readily applied.

## Background

Deciphering mechanisms of gene regulation is currently one of the key problems in molecular biology. The number of sequenced and annotated genomes is increasing rapidly, but we do not fully understand the regulatory networks underlying gene regulation. A few datasets approaching a genome-wide understanding of gene regulation in relatively simple organisms such as *E. coli *[1] or *S. cerevisiae *[2] exist, but especially for higher eukaryotes our understanding of gene regulation is far from complete. Experimental reconstruction of regulatory interactions is possible for relatively small systems [3], but it is impossible to scale this approach to all the available genomes. Therefore, computational methods are currently the best tool for improving our understanding of genome-wide gene regulation.

### Biological background

The process of transcriptional regulation is facilitated by proteins called transcription factors which bind to DNA sequences to help or prevent the initiation of transcription by RNA polymerase. This binding is selective, i.e. trans-factors bind only to specific DNA sequence motifs (called cis-elements) [4]. In higher eukaryotes, many genes need to exhibit complex spatio-temporal expression patterns. The key to achieving such complexity is the combinatorial transcription regulation [5], i.e. different combinations of similar *cis*-elements may yield different expression profiles. Sequence elements, whose main function is driving complex expression patterns, are often referred to as *cis-regulatory modules *(CRMs). Throughout this paper, we will use this term, but it should be noted that our method is limited to finding CRMs that are relatively close the transcription start site (TSS) of a gene of interest (in the range of 10 kb up- or down-stream of its TSS) whereas in general the term "CRM" may also be used to refer to distant enhancers which cannot be found using our method.

### Previous work

The earliest computational approaches to discovering CRMs in non-coding DNA were based on two observations:

• CRMs contain unusually high concentration of binding sites [6],

• CRMs are more conserved across species than other non-coding sequences [7].

These early approaches sparked a number of studies which utilize different computational approaches to find CRMs based on these two presumed properties [8-19]. However, in the light of more recent analyses of the statistical properties of CRMs [20], neither assumption appears to be a reliable foundation for CRM prediction. After analyzing over 500 experimentally verified CRMs from *D. melanogaster*, Li et al. claim that the clustering of motifs may reliably predict only a few CRMs (most notably the ones involved in the early blastoderm formation). Similarly, evolutionary conservation of CRMs appears to be less stringent and much more nuanced than previously thought. Firstly, CRMs are significantly more conserved than the rest of non-coding DNA only if measured by the density of short (7 bp) blocks conserved between species, rather than by simple sequence identity over larger windows. This is supported by recent findings that the evolution of CRMs is driven by gain and loss of whole binding sites rather than point mutations [21]. Secondly, even though the set of investigated CRMs was statistically conserved, the authors conclude that most CRMs are not distinguishable from other non-coding sequences based solely on conservation. These findings are not specific to *D. melanogaster *and are supported by a very recent study [22] based on comparing TF binding signatures in human and mouse liver.

However, there are two published studies addressing these issues at least partially. Hallikas et al. [23] propose the EEL algorithm for finding alignments of significant motif occurrences instead of the sequences themselves. This method is very efficient and does not rely on raw sequence similarity but it assumes that the motifs in conserved CRMs occur exactly in the same order. On the other hand, the BLISS method [24] approaches the same problem by analysis of a matrix containing occurrences of all motifs along both homologous sequences after Gaussian smoothing. This relaxes the assumption of conserved motif occurrence order but at the very high cost of computations. These two approaches fall into the category of *non-tissue-specific *methods. The approach reported in the present paper also falls into this category. The other group of methods, which could be called *tissue-specific*, are tuned for a particular type of CRMs, using either a set of several known specific motifs [9], or by learning such motifs from the known tissue-specific CRMs [8].

### Contributions of the present paper

We present a novel approach to finding CRMs in non-coding sequences associated with homologous genes. It is based on a simple method of scoring likelihood of the occurrence of a conserved combination of binding sites in a fixed-size window. This measure is constructed in such a way that it does not rely on strict criteria for neither sequence conservation, nor for motif clustering. We show that we are able to use the same parameters to discover motifs in human, rat, mouse and fruit fly using a universal, non-tissue-specific set of known motifs.

The overall procedure of the proposed method may be divided into the following steps:

• Finding occurrences of transcription factor binding site motifs obtained from a database in a set of DNA sequences proximal to transcription start sites of genes of interest.

• Calculating the alignment scores of motif sets in windows of fixed size using a novel method for homologous sequences from related species

• Measuring the rarity score of best alignments against a randomized set of promoters from the same species to filter out non-specific alignments.

The output of this procedure is a ranking of alignments of sequences along with the motifs contributing to this alignment. In the following section, we describe each of these steps in detail.

## Methods

### Identifying motif occurrences

In the present study we use models of transcription factor binding sites from the JASPAR CORE database [25]. It consists of 138 non-redundant motifs represented by frequency matrices. Each matrix has to be assigned a threshold of the log-likelihood of binding site affinity.

As explained in the following sections, our method of CRM identification takes into account both positive and negative signals from the promoter sequence. Thus, the control of both error types in the motif prediction has to be balanced, in the sense that the number of false positives should be of the same order of magnitude as the number of false negatives. As proposed by Rahmann et al. [26] we choose thresholds maintaining the balance between the control of type I and type II errors (see supporting materials for details).

In order to identify occurrences of a motif, sequences are scanned for words with log-likelihood above the related threshold.

### Comparing window contents

As discussed earlier, the presented approach is based on a slight relaxation of the motif order in the promoter region. We incorporate this idea by utilizing the concept of a fixed-size window with the assumption that the order of motifs which occur within the window does not matter, i.e. we treat the motif occurrences in one window as forming a multiset rather than a sequence. For this, we introduce a parameter *W *denoting the window length. We say that a motif occurs in a window if its left border occurs in that window. In order to compare contents *X*, *Y *of two windows we reward the common motif occurrences and penalize motifs which occur in one window but not in the other, as well as windows with empty motif sets. This leads to the following cost function:

*c*(*X*, *Y*) = *α*·|*X *∩ *Y*| - *β*·|*X *÷ *Y*| - *γ*,

where |*X *∩ *Y*| denotes the number of motif occurrences which are in common (intersection), while |*X *÷ *Y*| is the number of motif occurrences in one window but not in the other (symmetric difference). If a motif *M *occurs *M*_*X *_times in *X *and *M*_*Y *_times in *Y*, then its contribution to the term |*X *∩ *Y*| is min(*M*_*X*_, *M*_*Y*_), while its contribution to the term |*X *÷ *Y*| is |*M*_*X*_* - M*_*Y*_|. The constants *α*, *β*, *γ *are parameters of the cost function. We assume that *α *> 0 to give preference to windows containing common motif occurrences. Since multiplying the cost function by a positive constant does not change the relative assessment of the window content, it follows that we may assume, without loss of generality, that *α *= 1. Then, the role of *β *> 0 is to penalize for motifs occurring in one of the sequences but not in the other. It follows from our experiments that in case of a general motif database *β *should be much smaller than 1. The role of *γ *> 0 is to penalize pairs of windows with empty content which cannot be affected by changes of *α *and *β*. In our experiments we have verified that *γ *should be close to 0 and, in general, increasing it overly decreases the sensitivity of the method. For details of *β *and *γ *estimation see the section on Parameter estimation – case study of muscle specific CRMs.

**Figure 1:**
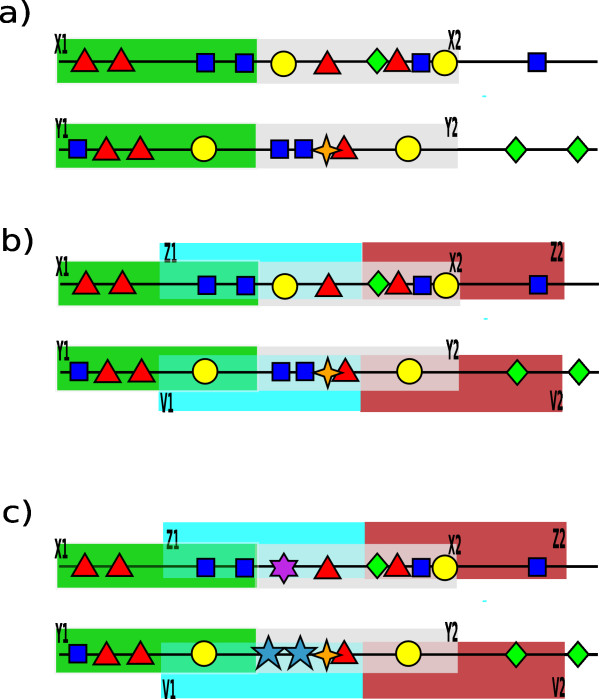
**Comparing window contents**. Part (a) shows two pairs of non-overlapping windows. Here we have *c*(*X*1, *Y*1) = 3 - 2*β *- *γ *and *c*(*X*2, *Y*2) = 3 - 4*β *- *γ*. Observe that motif occurrences near the border between the windows show high similarity. In part (b) we show two extra pairs of windows which partly overlap the windows from (a). The cost of extra windows is *c*(*Z*1, *V*1) = 4 - *β *- *γ *and *c*(*Z*2, *V*2) = 2 - 3*β *- *γ*. In part (c) there is presented a similar situation, except the motifs near the border have changed. We obtain the cost for the new contents: *c*(*Z*1', *V*1') = 1 - 7*β *- *γ *and *c*(*X*2', *Y*2') = 3 - 6*β *- *γ*. The other contents in (c) remain the same as in (b). Hence, the total cost of alignment in (c) is smaller by 3 + 8*β*.

Given two promoter sequences *S*_1 _and *S*_2_, we are looking for window stretches of the same length: one in *S*_1 _and one in *S*_2_, so that the cumulative cost for consecutive pairs of windows yields an "unusually high" score. Computing the score is illustrated in Fig. 1(a) where the total cost for two pairs of windows is 6 - 6*β *- 2*γ*. However, it may happen, as shown in Fig. 1(a), that the border between two windows may cut through an area of the promoter with high similarity of occurring patterns of motifs. In this case we have in Fig. 1(a) two occurrences of one motif (square) and one occurrence of another motif (circle). In order to accommodate for this situation we allow windows to overlap, i.e. we introduce a step *J *and assess fragments of promoter sequences as seen from the contents of a window which moves every *J *nucleotides. This is illustrated in Fig. 1(b) where *J *is taken as a half of *W*. In this case, the total score of the fragment becomes 12 - 10*β *- 4*γ*. On the other hand, when the border area between two windows does not show high similarity, the additional (shifted) windows contribute less to the total score as shown in Fig. 1(c). In this case the total score is 9- 18*β *- 4*γ*, which is smaller than the former score by 3 + 8*β*.

In order to reduce computation time we consider only window positions starting from the left end of a promoter sequence and jumping every *J *nucleotides. For a promoter sequence of length *L *this yields about *L/J *positions to investigate, instead of *L *positions if we considered all possible window positions. Also the smaller *J *is, the more window positions we have to consider. As changing the length of *J *produces only quantitatively different, but qualitatively results (see Additional file 1) we set the *J *parameter in our method to be half of the window length.

### Finding optimal window alignments

The next step is to find contiguous stretches of window pairs with maximal accumulated cost. Let us recall that we are considering two sequences *S*_1 _and *S*_2 _which are promoter regions for genes *g*_1 _(in species *A*_1_) and *g*_2 _(in species *A*_2_). We assume that *g*_1 _and *g*_2 _are homologs. Let the window length *W *and step *J *be fixed. Starting from the first position in each of the regions we move the window every *J *nucleotides until we reach the end of the region. Let *N*_1 _(resp. *N*_2_) be the number of such window positions in *S*_1 _(resp. in *S*_2_). For each 1 ≤ *i *≤ *N*_1_, let *X*_*i *_denote the contents of the window which starts in *S*_1 _at window position *i*. Similarly, let *Y*_*i *_be the contents obtained for *S*_2 _(for 1 ≤ *i *≤ *N*_2_). We build a *N*_1 _× *N*_2 _matrix *V *which stores in entry (*i*, *j*) a maximal value of the sum along the diagonal of the cost matrix *c*, which ends in position (*i*, *j*). More formally *V *(*i*, *j*) is computed as follows

$$V(i,j)=\{\begin{array}{ll} \max[0,c(X_{i},Y_{j})+V(i-1,j-1)] & \text{if }i,j>1\\ \max[0,c(X_{i},Y_{j})] & \text{otherwise} \end{array}$$

Having computed the matrix *V*, we are ready to do a pre-selection of CRMs. For each *i *≤ *N*_1 _let *j *≤ *N*_2 _be the index such that *V *(*i*, *j*) is maximal among all the values in the *i*-th row of *V*. Let (*i*, *j*), (*i *- 1, *j *- 1),...,(*i *- *k*, *j *- *k*) be the maximal contiguous fragment of a diagonal of *V *which consists of a strictly decreasing sequence of values in V. A CRM pre-selected in *S*_1 _is the region from window position *i *- *k *through *i *(recall that positions in *V *correspond to window positions in the promoter sequences). At this stage we declare the CRM preselected since we have to further assess the likelihood of its occuring in the genome by chance.

### Assessing the rarity of CRMs

Even though the most natural guideline for selection of CRMs is the accumulated cost of the pre-selected CRMs, we have to adjust this statistic in order to avoid a bias due to possible species-specific abundance of "random" occurrences of certain motifs. In the present paper we propose a simple heuristic approach to this issue, considering computational feasibility of the whole method. It is based on the idea that CRM in principle should be specific to some group of genes, but not too abundant in the genome. If not accounted for that, the results tend to contain mostly results from the core promoter area and from repetitive elements. Since it is not desirable to filter out these sequences (particularly filtering out core promoters could lead to serious problems), we set out to propose a quantitative measure of alignment *rarity*, which is supposed to promote specific alignments. The approach we take stems from simulation methods for obtaining random promoters, but since we need to retain the features of relatively long stretches of sequences, instead of randomly shuffling the sequences, we select a sample of random promoters from the considered genome and calculate how often the aligned region gets an alignment score at least as high as when it is aligned with its homologue. If this occurs frequently we consider such an alignment not interesting as a putative CRM.

We describe this procedure of randomization little more precisely. First of all our approach assumes that the user knows which area of promoter sequence is of interest to her/him. For instance, in our experiments with the muscle and liver genes we consider the promoter area to be -10 kb through +5 kb of the start of transcription, but other values can be considered as well. In order to assess the rarity in a given experiment we sample a set of 99 genes from the genome of the other organism and take their promoter regions which are of the same size and similar position with respect to the start of transcription as assumed by the approach. Then, for each gene g whose predicted CRM rarity we want to assess, we compute the score of g against each of the above mentioned 99 promoters. For a given position in the matrix, instead of the raw score *V *(*i*, *j*), we consider the position of this score in the ranking of 100 sequences (and hundred matrices). This gives us an estimate of the relevance of this prediction. Detailed procedure is described in the following paragraph.

Let us keep the notation of the previous section (i.e. the matrix *V*, the genes, *g*_1 _and *g*_2_, the sequences *S*_1_, *S*_2_). We first explain how we compute the *S*_1_-*view rarity of *(*i*, *j*), for a given position (*i*, *j*) in *V*. We retrieve 99 randomly selected sequences *R*_1_,...,*R*_99 _which are promoter regions of genes in species *A*_2 _such that none of the genes is homologous to *g*_2_, and each sequence *R*_*m *_is of the same length as *S*_2 _(i.e. of length *N*_2_). Next, for each *m *= 1,...,99 we build the accumulative cost matrix *V*_*m *_by computing window contents of *S*_1 _against those from *R*_*m*_. Matrix *V*_*m *_is computed in the same way for *S*_1 _and *R*_*m *_as *V *was computed for *S*_1 _and *S*_2_. For *m *= 1,...,99, let *v*_*m*_(*i*) be the maximal element in the *i*-th row of *V*_*m*_. Let *p *be the position of *V *(*i*, *j*) in the set {*v*_1_(*i*),...,*v*_99_(*i*), *V*(*i*, *j*)} counted in ascending order. *Ex aequo *occurrences of *V *(*i*, *j*) with the other values are resolved to the benefit of these other values, i.e. *V *(*i*, *j*) always occupies the last position in the block of equal values. We set *S*_1_-view rarity of (*i*, *j*) as the ratio *p/*100.

Assume we want to assess rarity of a preselected CRM in promoter sequence *S*_1 _and assume that this CRM was obtained from the fragment of *V *for positions (*i*, *j*), (*i *- 1, *j *- 1),...,(*i *- *k*, *j *- *k*). The rarity of this CRM in *S*_1 _we define as the minimum *S*_1_-view rarity of these positions.

### Evaluating the quality of CRM predictions

In order to assess the performance of any CRM prediction method one has to choose a proper objective function. In this paper, we test our method on the data with known CRM data. It can be viewed as a classical prediction problem and scored with measures of sensitivity and specificity [9]. However, two facts should be accounted for in the case of CRM prediction:

(i) the expected number of negative examples is by far greater than the expected number of positive ones. For example, in our experiments for each promoter sequence in the muscle set, there are 300 windows of size 100, out of which only a few comprise CRMs. Similarly, Philippakis and Bulyk [9] consider 1000 negative examples for a dataset containing 27 CRMs (see Section on Comparison with other methods).

(ii) It is expected that there are more CRMs than the ones collected in the training dataset. For this reason, some "false positive" predictions might be actually true CRMs.

To account for the first problem, Chan and Kibler [27] propose one more measure, *positive predictive value *(PPV), which is the ratio of true positives among all predictions. They also note that, because of (ii) the values obtained for PPV are underestimates.

We believe that because of (i) any method based solely on the notions of true/false positives/negatives leads to misjudging the performance of CRM prediction. Specifically, if the number of negative examples is high compared to the positive ones, a method returning many predictions is scored better than the one giving fewer results. We consider such methods impractical. Therefore, we use a very simple but useful measure of prediction quality based on the position of the correctly predicted CRM in the ranking of predictions returned by our program. Given a threshold *k*, we say that the CRM is *found*, if the correct prediction is among the top *k *predictions (we call a prediction correct if it overlaps with the CRM and is not longer than 3 times the length of the true CRM). As the *mean quality *of a prediction method for a set of sequences annotated with known CRMs, we consider the ratio of found CRMs to the all known CRMs. The choice of *k *is arbitrary, but in real applications it should not be smaller than 5 because of (ii) and at the same time it should not be limited by the length of the sequence divided by the expected length of a CRM. We use the value *k *= 5 for mammalian datasets and *k *= 10 for fruit fly dataset (since there are 5 annotated CRMs in one promoter sequence, selecting only 5 top ranked alignments would be too restrictive). Another advantage of our scoring procedure is the fact that, as opposed to the PPV score, it is not sensitive to overlapping predictions. More precisely, generating many overlapping results for the high confidence regions may increase the PPV score of a method, it cannot increase the number of found CRMs. Even though we use our quality measure to optimize the parameters, we report in Section on Comparison with other methods the values of Sensitivity, Specificity and PPV. We also report the actual values of the overlap between our predictions and true CRMs (normalized by the sum of the lengths).

## Experimental results

For all experiments with biological data discussed here we chose the window length *W *= 100 and step *J *= 50. Other values of *W *in the interval 50 through 250, as well as other steps, gave similar results (data not shown).

Organization of this section is as follows: we optimize parameters for our method on a set of muscle specific CRMs which have been experimentally verified. Then we show that our method gives reasonable results for other CRMs with these parameters: liver specific CRMs in human and the CRMs for the *eve *gene in *D. melanogaster*.

### Parameter estimation – case study of muscle specific CRMs

We estimated the appropriate parameters on a large set of muscle specific CRMs reported by Wasserman and Fickett [28]. It consists of 43 CRMs, mainly from human, mouse and rat. After removing from the list of CRMs those coming from other species (chicken, hamster, pig, cow) the remaining CRMs, 37 in total, were manually checked and verified at Ensembl database [29]. This step was essential since the original data was outdated with respect to the current genomes deposited in the database. In some cases we were not able to map a CRM to the corresponding gene. After omitting these doubtful cases we were left with 24 CRMs corresponding to 23 genes (5 genes are from human, 12 from mouse and 6 from rat). One human gene (DESMIN) had two CRMs. For all aforementioned genes and their homologs (in the other two species) we retrieved promoter regions flanking from -10 Kb through +5 Kb relative to the TSS according to Ensembl. We thus have created 48 pairs of promoter regions corresponding to homologous genes. The choice of the sequence length here was a trade-off between covering as many CRMs from [29] and running time of the learning procedure.

**Figure 2:**
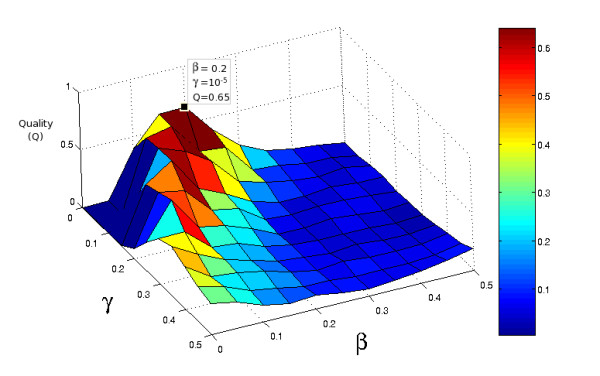
**Prediction quality as a function of *β *and *γ *parameters**. The prediction quality (Q) for the training (muscle) dataset is plotted here as a function of the *β *and *γ *parameters. The maximum value is marked by a square. It should be noted, that the prediction quality seems to be close to zero for most values except for the small area around maximum. Our experiments with wider ranges of parameters (data not shown) also support that hypothesis.

Estimation of parameters was performed on a grid of values for *β *and *γ*. We first examined the intervals 0 through 2 for both parameters with step 0.2 (in fact, we replaced 0 with 1·10^-5 ^due to the considerations in section on comparing window contents). For each set of parameters we computed the mean quality of CRM prediction (see subsection on evaluating the quality of CRM predictions). After the area with an optimal score was localized, we performed again the estimation on intervals 0 through 0.5 with step 0.05 for both parameters (as above replacing 0 with 1·10^-5^). A plot of the obtained prediction evaluations is shown in Fig. 2. The best results were obtained for *β *= 0.2 and *γ *= 1·10^-5^. A set of more detailed numerical results is given in the supporting material.

**Figure 3:**
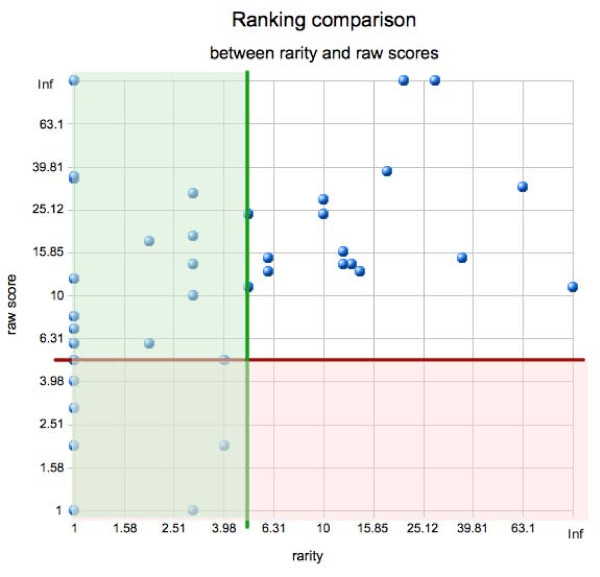
**Comparison of the raw score and the rarity score rankings**. The plot shows the comparison of rankings obtained for the CRMs from the training set using raw score vs. rarity scores. Each point corresponds to a single CRM. The position of the dot depends on the ranking of this CRM using raw scores (X-axis, log scale) and its ranking according to the rarity score (Y-axis, log scale). The green and red lines are placed at *k *= 5 for rarity and raw scores respectively. The points placed below (red shaded area) and left (green shaded area) of these lines are considered to be found by respective methods. As we can see, there are no points found only when using the raw score but substantial number of them is found only when using the rarity score. It should be noted, that the parameter estimation was done for both rankings separately, i.e. optimal parameters *β *and *γ *were used for both methods. The data for this table is available in Additional file 1.

An important question is whether the introduction of the rarity score improves the performance of the method. In order to verify that, we have repeated the parameter fitting procedure using only the raw alignment scores without computing the rarity. The optimal parameters were in fact slightly different (*β *= 0.4, *γ *= 0.05), but the overall prediction quality dropped dramatically. We were able to predict only 1/3 of examples and it is worth noting that the rankings of the predictions are in 40 cases out of 50 cases lower. It is important that even though in some cases (6 out of 50) the raw score gives a better ranking, these are only in cases where the rarity score gives a prediction within the top 5 as well. However, in 23 cases the correct prediction is ranked among the top 5 by the rarity score, whereas the raw score is below that cutoff. The comparison of the two rankings is presented in Fig. 3. All results of our method on the training dataset are presented in Additional file 1.

### Prediction of liver specific CRMs in human

The experiment was performed on the set of liver specific CRMs reported by Krivan and Wasserman [30]. The dataset consists of 16 CRMs: 10 from human and 6 from other species. We have manually selected 7 human CRMs which, according to Ensembl, lie in the regions from -10 Kb through +5 Kb relative to the TSSs. Then, we retrieved the flanking sequences for the selected genes and their homologs in rat and mouse. Both these species have two homologs of human insulin and one homolog for each of the other genes which gives 16 gene pairs altogether.

The algorithm was run on all 16 pairs of homologous promoter regions with the parameters chosen for the muscle specific data in the previous section (*β *= 0.2 and *γ *= 1·10^-5^). The result of CRM prediction is reported in Table 1 (a more detailed version is included in Additional file 1). Out of 7 CRMs, 4 are clearly predicted (rarity ≤ 0.1, ranking ≤ 5) and the remaining 3 are not found (rarity ≥ 0.8). This result is comparable with the performance of the method on the training dataset. Observe that the well predicted CRMs have a significant overlap.

**Table 1:** Prediction quality of liver specific CRMs in human.

human gene	homolog species	prediction
ALDOB	mouse	incorrect
	rat	incorrect

IGF1	mouse	correct
	rat	correct

PAH	mouse	incorrect
	rat	incorrect

PROC	mouse	correct
	rat	correct

CYP7A1	mouse	incorrect
	rat	incorrect

G6PC	mouse	correct
	rat	correct

INS	mouse	incorrect
	mouse	correct
	rat	correct
	rat	correct

For each gene name in column 1, one row shows CRM prediction for one homolog (described in column 2).

If the most significant prediction which overlaps the experimentally verified CRM is in the top 5 in the rarity ranking, we call it correct, and incorrect otherwise. An extended version of this table can be found in Additional file 1.

### Predicting even-skipped CRMs in fruit fly

**Figure 4:**
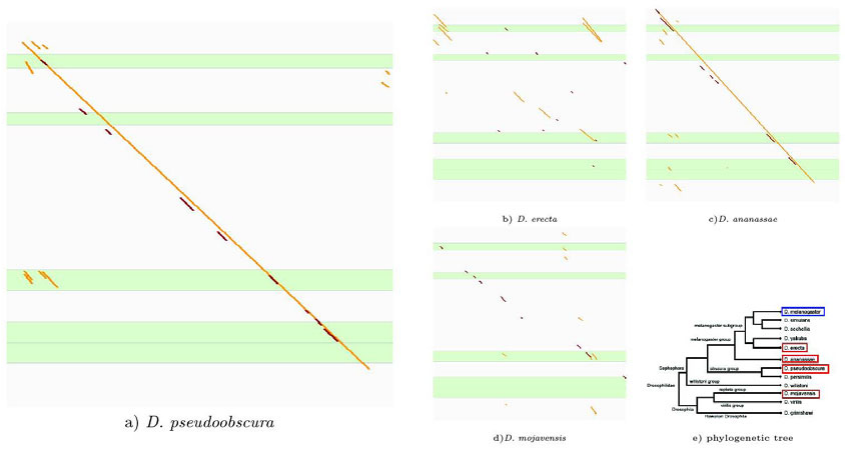
**Comparison of CRM predictions in even-skipped gene in different Fruitflies**. In parts (a) through (d) the Y-axis represents positions in the promoter region of *eve *gene of *D. melanogaster *with 5' end at the top and 3' end at the bottom, while the X-axis represents positions in the promoter region (with 5' end corresponding to the left and 3' end corresponding to the right end) of *erecta, ananassae *and *mojavensis*, respectively. Brown strips represent the top ten predictions by our method, while orange strips represent the top ten predictions by EEL. Light green horizontal areas represent positions of the experimentally verified even skip CRMs in *D. melanogaster*. Part (e) presents a phylogenetic tree of 12 *Drosophila *species including the ones discussed here.

In this experiment we have concentrated on *cis*-regulatory modules for the well studied gene *eve *in *Drosophila melanogaster*. We extracted the relative positions of the following five experimentally verified eve CRMs from REDfly database [31]: eve_stripe1, eve_stripe2, eve_stripe4_6, eve_stripe5, and eve_stripe_3+7. Their length ranges from 500 to 800 nucleotides. The promoter regions corresponding to the eve gene in (several species) were downloaded from Flybase [32]. The reason for taking the region (-5 Kb, +10 Kb), rather than (-10 Kb, +5 Kb) was in order to make sure that the five CRMs of interest are included in the selected area while keeping the same overall sequence length. These four Drosophila species have diverged from *D. melanogaster *so that *D. erecta *and *D. melanogaster *are the closest relatives, while *D. mojavensis *and *D. melanogaster *are evolutionarily furthest apart among these four. Fig. 4(e) contains a phylogenetic tree for these five flies together with other seven *Drosophila *species.

We ran our method on four pairs of promoter regions with the same parameters as for other datasets (*β *= 0.2 and *γ *= 1·10^-5^). The quality of prediction of the experimental CRMs as retrieved by our method are shown in Table 2. With the exception of stripe_5, all other CRMs were predicted correctly for the pair *D. melanogaster/D. pseudoobscura*. The results for *D. melanogaster/D. ananassae *pair were marginally worse than for the previous pair. The results were not satisfactory for the closer relative *D. erecta *nor for the farthest relative *D. mojavensis*. This could be explained by the fact that evolutionary distance between *D.mel*. and *D.pse*./*D.ana*. is similar to the distance between human and mouse/rat. This suggests that a reference species should be selected so that the evolutionary distance is similar to that in the training dataset. Though we did not investigate this issue because of the lack of proper training dataset, it is possible that we could get better predictions of even skip CRMs for a different set of parameters for different evolutionary distances. Nevertheless, it is remarkable that despite applying parameters that were estimated for different species and for genes with different tissue specificity we obtained predictions of similar quality.

**Table 2:** CRMs in the gene eve in fruit fly.

CRM homolog	*Drosophila erecta*	*Drosophila ananassae*	*Drosophila pseudoobscura*	*Drosophila mojavensis*
stripe3+7	-	-	+	+

stripe2	-	-	+	+

stripe4 6	-	-	+	+

stripe1	-	+	+	+

stripe5	-	-	-	-

The table reports quality of the most significant prediction of each CRM in the gene *eve *in *Drosophila melanogaster *obtained by our method with each of other considered *Drosophila *species. The key to values is the same as in Table 1, however since we are looking for 5 CRMs, we have adjusted the value k to 10.

Fig. 4 presents the top ten predictions for four pairs of flies obtained with our method. They are presented by brown stripes (orange stripes are included in this figure for comparison with another method, see the next section). The reader may notice that in addition to the sought after experimental CRMs there are a number of new putative CRMs which may be false positives, but may turn out to be true *cis*-regulatory modules. Some of the putative CRMs are supported by more than one pair which may be an indication of a true CRM.

The set of motifs used in this study was a broad spectrum of motifs from JASPAR CORE. We have also investigated the impact of choosing more specific motifs for our approach. We have run the experiment for a set of eight specific motifs which constitute the even skip CRMs. However, the results were rather discouraging (data available in Additional file 1), suggesting that the general purpose motifs such as JASPAR CORE are better suited for discovering evolutionarily conserved CRMs. One possible explanation for this observation is that such species-specific motifs are ubiquitously conserved in the fly genome and therefore true CRMs do not stand out in comparison with the background model. We therefore propose that JASPAR CORE (or a similar set of non-species-specific motifs) may be a better choice for predicting conserved CRMs.

### Comparison with other methods

Recall that methods available for the task of CRM prediction can be divided into two distinct classes based on the chosen approach:

• tissue-specific methods, tuned for a particular type of CRMs, using either a set of several known specific motifs [9], or learning such motifs from the known tissue-specific CRMs [8],

• general methods based on a universal motif set (e.g. [23,24]).

The method proposed in the present paper belongs to the second class and our comparison is carried out within this class. We refer the reader to [8] for a thorough comparison of the performance of tissue-specific approaches.

There are two published methods proposing computational prediction of CRMs based on a non-specific motif set. Though we could not compare our results with the BLISS algorithm [24], as it seems to be limited to sequences significantly smaller than 15000 bp. We believe that this is due to the fact, that the computation cost was too high. Actually, the time complexity of BLISS algorithm is O(*L*^2^*m*), where *L *is the promoter length and *m *is the number of considered motifs. On the other hand the complexity of our method is O(*L*^2^) (see Additional file 1). Even though the asymptotic complexity of these methods with respect to *L *is the same (if we consider *m *as a constant), the number of atomic operations which have to be performed by our method for the actual values of *L *and *m *is approximately 250 thousand times smaller than for the BLISS method. This is in part due to using 'step' by our method. It should be noted that the running time of our method is less than a minute for a pair of sequences of length 15*kb *on a standard workstation PC.

Computation time is also a strong point of EEL software [23], which is available for download. We have rerun the EEL software on the same datasets as our method. For the muscle dataset, it was able to recover altogether 14 of the 24 CRMs (detailed results are in Additional file 1). For the liver dataset, the results of the two methods were comparable: EEL was able to recover 5 out of 7 CRMs, while we were able to recover 4 from the same data.

The results for the fruit fly dataset are presented in Table 3. We present here the performance of both methods in recovering the even-skipped gene CRMs using the homology with *D. pseudoobscura*. The values of sensitivity and PPV are presented in two variants: assuming top 5 predictions or top 10. Another view of the same results is presented in Fig. 4. We present there top 10 predictions for both methods for all 4 pairs of homologous sequences. In three cases (a, c, d) our method provides reasonable predictions while the EEL method is often providing wrong predictions. It should be noted that a possible reason for poorer performance of EEL on the Fruifly dataset is that the set of parameters used by EEL for mammalian genomes cannot be used to the analyses on insect genomes, even though the authors of EEL [23] show that it is able to recover the same enhancers of *eve *with a specific set of motifs (and possibly different parameters, however this is not clear from their study). In contrast, the parameters for our method seem to be applicable to Fruifly data, especially when comparing *D. melanogaster *to *D. pseudoobscura*.

**Table 3:** Prediction quality for fruit fly CRMs. The table reports prediction quality for CRMs in *Drosophila melanogaster *obtained with *Drosophila pseudoobscura*.

	our method			EEL		
Top rank	found	SN	PPV	found	SN	PPV

5	2	0.4	0.4	1	0.2	0.2
10	4	0.8	0.4	2	0.4	0.2

## Discussion and conclusion

The novel method of predicting *cis*-regulatory modules which is proposed in the present paper is based on the following two salient features:

• implementation of a mixture of sequential and set-theoretic evaluation of similarity measure for groups of motif occurrences;

• introduction of a rarity measure for putative CRMs.

Both of these above features play an important role in the quality of CRM prediction. Introduction of a rarity measure for putative CRMs plays a crucial role in moving true positives up in the ranking. We propose a straightforward method of computing this rarity measure, having mainly computational efficiency in mind. Further research should clarify whether we can improve with respect to the quality of predictions when adopting less naive ways of computing rarity without affecting computational time. The main contribution and power of the present method lays in combining both: sequential and set-theoretic aspects of assessing similarity of motif clusters. As mentioned earlier one way of approaching the problem of predicting CRMs is via discovering conserved non-coding sequences and then finding their subsequences that contain a large number of motif occurrences. This is partly covered by our method, since if two promoter fragments have a similar sequence and are drawn from the same background, then the penalty for symmetric difference of sets of motif occurrences in these fragments is zero, and what really counts is the number of such occurrences. On the other hand, in our method we relax the assumption of sequence conservation since we are working solely with motif occurrences. We also do not assume a strict conservation of the order of motif occurrences by allowing disruptions of this order without any penalty, provided that the occurrences are not too far apart, i.e. they fit into one window. For the same reason we do not penalize differences in relative distances of motifs, providing the differences are within the window length.

Also, even though in our analyses all known CRMs considered are of comparable size (mostly 100–500 bp), the range of possible sizes of CRMs is still debated. Judging by the sizes in databases of CRMs, the regions are often much larger (even up to 5 kb, see [31]), while one of the most well studied enhanceosomes (Iterferon-*β*) is only 60 bp in length [33]. Given this wide range of possible CRM lengths it may be considered an advantage that our method puts less constraints on the CRM length as other, most notably conservation-based, methods.

An appealing feature of our method is that it is tailored for use with a non-specific set of motifs. The results of experiments presented here show clearly that a non-specific set such as JASPAR CORE works very well for muscle and liver, as well as for the even skip CRMs.

Another important issue which emerges from the results of our method is that the quality of CRM prediction may largely depend on the evolutionary distance of a relative organism against which we compare constellations of CRMs. As we have seen, when the species of interest is *D. melanogaster*, we obtained unsatisfying results when the chosen relative was *D. erecta*. The outcome was better for *D. ananassae*, best for *D. pseudoobscura*, but again worse for *D. mojavensis *(but not as bad as for *D. erecta*).

It should be noted that *D. pseudoobscura *and *D. ananassae *are in a similar evolutionary distance to *D. melanogaster *as rat and mouse are to human [34] It also seems that the set of relatives mouse/rat works well for human.

### Parameters

In total our approach uses the following four internal parameters. Remarks on selection of these parameters are given in section on experimental results.

• *W *– length of the window (set to 100);

• *J *– length of step (set to 50);

• coefficients in the cost function (as discussed in the text the parameter *α *can be set to 1 without loss of generality):

(i) *β *– penalty for difference in motif composition (set to 0.2);

(ii) *γ *– CRM extension penalty (set to 10^-5^).

In order to filter the obtained results the user may choose the following two parameters:

• The threshold rarity (we use 0.05);

• the number *k *of top predictions to be displayed (we used *k *= 5 for liver and muscle data, and *k *= 10 for the gene even-skipped).

It should be noted that the total length of promoter sequences, as well as the position of left and right flank with respect to the start of transcription can be also considered a parameter, since the user may choose to search for CRMs using flanking regions of different size and different relative position.

## Availability and requirements

Project name: Billboard

Project home page: 

Operating system(s): Platform independent (tested only on Linux)

Programming language: Java (requires JDK6 and the Ant tool)

License: GNU GPL

Any restrictions to use by non-academics: None

## Abbreviations

CRM: cis-regulatory module; PPV: positive prediction value.

## Authors' contributions

BW, JT and ND conceptualized the study and wrote the paper; All authors read and approved the manuscript; JT proposed the approach based on windows and the score function; BW implemented a prototype, proposed the rarity measure and performed the experiments; ND devised the dynamic algorithm for adaptive motif thresholds; MP implemented the final software.

## Supplementary Material

Additional file 1 **Supplementary Materials.** Supplementary Materials include description of some technical details (computing thresholds of motif occurences, impact of window size and step on training quality) some result details (tables, figures, ROC curves) and a note on computational complexity. Click here for file
