# Supplementary material for: Finding evolutionarily conserved cis-regulatory modules with a universal set of motifs
Source: BMC Bioinformatics. 2009 Mar 10;10:82. doi: 10.1186/1471-2105-10-82 (PMC2669485; doi:10.1186/1471-2105-10-82)
Supplement: Additional file 1 — Supplementary Materials. Supplementary Materials include description of some technical details (computing thresholds of motif occurences, impact of window size and step on training quality) some result details (tables, figures, ROC curves) and a note on computational complexity. [file 1471-2105-10-82-S1.pdf]

Finding evolutionarily conserved *cis*-regulatory modules with a universal set of motifs – Supplementary Materials

Bartek Wilczyński, Norbert Dojer, Mateusz Patelak and Jerzy Tiuryn

November 6, 2008

1 Computing thresholds for motif occurences

For each motif M, all $|M|$-subwords w of a sequence are scored with the function

$$L_M(w) = \log \frac{P(w|M)}{P(w|B)}$$

where $P(w|M)$ is the probability of observing w given the motif model (drawn from the frequency matrix) and $P(w|B)$ is the probability of observing w given the background model (estimated from the sequence). All subwords w satisfying $L_M(w) > t_M$ are classified as M-occurrences.

There are two standard approaches to the choice of the threshold t_M [1]. The first one aims at restricting the number of false positive motif occurrences. For assumed type I error level α_1, t_M is chosen to satisfy $P(L_M(w) > t_M|B) = \alpha_1$. Its disadvantage is poor control on the classification of true M-occurrences.

The second approach (setting t_M satisfying $P(L_M(w) < t_M|M) = \alpha_2$ for assumed type II error level α_2) restricts the number of false negatives. Unfortunately, it leads to the loss of control on the number of false positives, and consequently to significant disparity in the number of predicted instances of strong and weak motifs (i.e. motifs easily and hardly discriminated from the background).

As it is explained in the main text, our method of CRM identification takes into account both positive and negative signals from the promoter sequence. Thus the control of both error types in the motif prediction has to be balanced, in the sense that the number of false positives should be of the order of the number of false negatives. Following the approach proposed by [1] we set the threshold t_M satisfying the equation

$$P(L_M(w) < t_M|M) = 1000 \cdot P(L_M(w) > t_M|B)$$

The constant 1000 is chosen due to the fact that the average number of binding sites of a motif is three orders of magnitude lower than the number of all positions (see e.g. the considerations of [2]).

The problem in computing the threshold t_M lies in finding the distributions of L_M under the background and the motif model.

Given motif M with a Position Weight Matrix $(M_i(x))^{1\le i\le |M|}_{x\in\{A,C,G,T\}}$ the scoring function L_M decomposes into the sum of respective PWM coefficients:

$$L_M(x_1 \dots x_{|M|}) = \sum_{i=1}^{|M|} M_i(x_i)$$

Let $P_i(l|B)$ denote the probability of observing under the background model some word $x_1 \dots x_|M|$ satisfying $\sum_{j=1}^{i} M_j(x_j) = l$. We have

$$P_0(l|B) = \begin{cases} 1 & \text{for } l = 0 \\ 0 & \text{otherwise} \end{cases}$$

and for $i > 0$

$$P_i(l|M) = \sum_{x\in\{A,C,G,T\}} P_{i-1}(l - M_i(x))P(x|B)$$

Similarly, for $P_i(l|M)$ denoting the probability of observing under the motif model some word $x_1 \dots x_{|M|}$ satisfying $\sum_{j=1}^{i} M_j(x_j) = l$, we have

$$P_0(l|M) = \begin{cases} 1 & \text{for } l = 0 \\ 0 & \text{otherwise} \end{cases}$$

and for $i > 0$

$$P_i(l|M) = \sum_{x \in \{A,C,G,T\}} P_{i-1}(l - M_i(x))P(x|M(i))$$

where $P(x|M(i))$ is the probability of observing x on i-th position under the motif model.

Finally, the distributions of the score L_M under the background and under the motif model are given by the probabilities $P_{|M|}(l|B)$ and $P_{|M|}(l|M)$, respectively.

Unfortunately, a dynamic programming algorithm computing score distributions based on the above equations has time complexity $\mathcal{O}(4^{|M|})$. Therefore we approximate the value of the threshold t_M with satisfying for our purposes precision $p = (l_{max} - l_{min})10^{-3}$, where l_{max} and l_{min} are maximal and minimal L_M values, respectively. It is done through rounding the coefficients to closest multiples of $\frac{p}{|M|}$ and, consequently, restricting the set of possible l-values. In this way the time complexity is reduced to $\mathcal{O}(\frac{|M|^2}{p})$.

2 Impact of window size and step on training quality

In order to verify whether the choice of window size has an impact on the prediction quality, we have evaluated our method prdictions quality on the training set using different window and step sizes. Since the window size should be greater than a size of a single motif and at the same time smaller than the size of the enhancer, we are effectively limited to a range between 20 and 200bp. When it comes to the step size, it should be smaller than the window size and it is very convenient to choose such a step size, that the window size is divisible by the step. For this reason, we have chosen the window sizes (W) equal to 24, 36, 48, 96, 144 or 196, and the step size of $W/2$, $W/3$ and $W/4$ for each W. We have repeated the training procedure for all these values of W and J and calculated the prediction quality, i.e. the fraction of true enhancers overlapping with one of the top five predictions, and and the average overlap of the predictions with true enhancers. The reults are plotted in the following figures. As we can see, the prediction quality is slightly increasing with the increase of the window size, but the average overlap starts decreasing above the window size of 100. The step does not seem to have a large impact on the measured quantities, so we have chosen $J = W/2$ which minimizes the cost of computations.

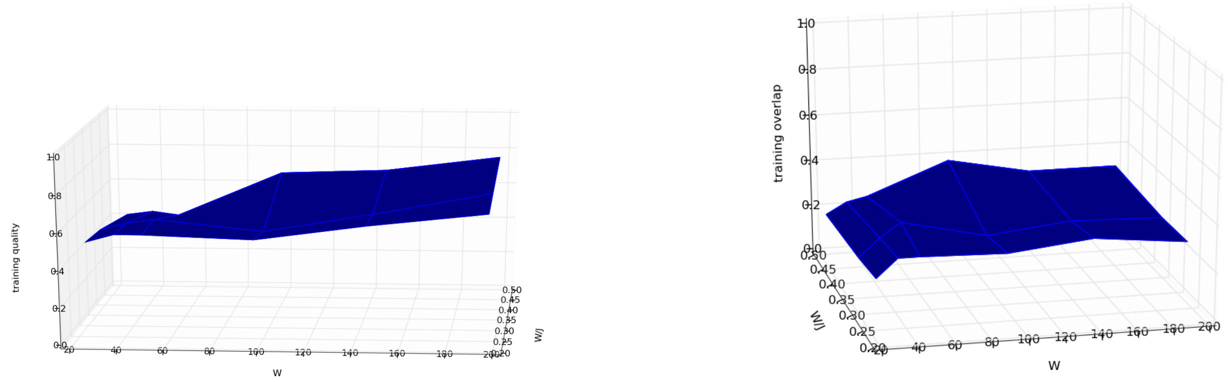

a) b)

Figure 1: Prediction quality (a) and overlap (b) as functions of window(W) and step(J) size.

3 Muscle specific CRMs in Mouse and Human

Table 1: Prediction quality of muscle specific CRMs in Mouse

mouse gene	homolog from human			homolog from rat		
	signif.	ranking	overlap	signif.	ranking	overlap
Ckm	0.27	13-14	0.78	0.11	10	0.68
Myh7	0.01	1-3	0.49	0.01	1-4	0.65
Myog	0.01	1	0.41	0.01	1-3	0.40
Tnnc1	0.01	1-3	0.50	0.01	1	0.65
Myf6	0.01	1	0.38	0.01	1	0.37
Myh6	0.01	1-5	0.51	0.02	3-5	0.72
Chrnb1	0.95	12-13	0.03	0.08	6-9	0.63
Chrng	0.01	1-6	0.36	0.03	2	0.45
Chrnd	0.51	63-68	0.49	—	—	0.00
Acta1	0.03	4	0.90	0.01	1-4	0.62
Chrne	0.01	1-3	0.78	0.01	1-8	0.85
Chrna1	0.89	36-39	0.47	0.26	10-11	0.22

Table 2: Prediction quality of muscle specific CRMs in Human

human gene and crm	homolog from mouse			homolog from rat		
	signif.	ranking	overlap	signif.	ranking	overlap
DES crm1	0.27	14-15	0.63	0.32	12	0.34
DES crm2	0.03	3	0.49	0.06	5	0.56
MYH7	0.03	3-4	0.73	0.18	21-23	0.56
MYOG	0.19	2	0.41	0.22	3	0.59
ACTC1	0.10	4	0.88	0.01	1	0.82
ACTA1	0.01	1-4	0.53	0.01	1-2	0.53

4 Importance of rarity score

In Table 3, we show the comparispon betwwen the raw and rarity-based rankings. Each line in the table corresponds to a pair of sequences in the training set. The left column contains the name of a corresponding gene. For each CRM there are two pairs of sequences corresponding to different homologues. The numbers in the second and third column are the rankings obtained by the true CRMs using the rarity score (column 2) or the raw alignment score (column 3). The lower the ranking, the better. If the ranking of the true CRM is not larger than 5, the CRM is considered to be found which is indicated by using bold face. If the true CRM was not reported in the ranking at all , we put ∞ instead of a number. It should be noted that the parameters β, γ were optimized for both rankings separately not to give any of the methods an unfair advantage.

Table 3: Comparison between raw and rarity score based rankings

Gene	Ranking using rarity	Ranking using raw score	Gene	Ranking using rarity	Ranking using raw score
ENSRNOG00000003777	**1**	35	ENSG00000175084	12	14
	28	∞		**5**	24
ENSG00000175084	14	13	ENSG00000197616	**3**	10
	**3**	19		21	∞
ENSMUSG00000030399	13	14	ENSRNOG00000004878	**1**	**4**
	10	24		**1**	**2**
ENSMUSG00000053093	**1**	**3**	ENSMUSG00000026459	**1**	**5**
	**1**	**1**		**1**	**2**
ENSMUSG00000021909	**1**	8	ENSG00000122180	**2**	6
	**1**	**1**		**3**	**1**
ENSRNOG00000017786	**1**	**2**	ENSRNOG00000025757	**1**	6
	**1**	**4**		6	15
ENSRNOG00000017226	**5**	11	ENSRNOG00000019602	**3**	30
	18	38		**4**	**5**
ENSG00000159251	**1**	**5**	ENSMUSG00000035923	**1**	**4**
	**4**	**5**		**1**	**3**
ENSMUSG00000040752	**1**	6	ENSMUSG00000041189	6	13
	**3**	14		12	16
ENSMUSG00000026253	**1**	∞	ENSG00000143632	**1**	8
	**2**	18		**1**	**4**
ENSMUSG00000026251	∞	11	ENSMUSG00000031972	**4**	**2**
	63	32		**1**	7
ENSMUSG00000014609	**1**	36	ENSMUSG00000027107	36	15
	**1**	12		10	28

5 Predictions of Billboard and EEL methods on the liver dataset

For reference, in Figure 2 we present top predictions of the Billboard and EEL methods on the liver dataset. In all plots, the x-axis represents the sequence and the bars correspond to different predictions by two methods against two organisms (Rat and Mouse). The highest, purple bar corresponds to the location of the reference enhancer. The height of the other bars corresponds to the prediction score relative to the maximum score (in case of billboard it is 1-rarity). The case of the ENSG00000129965 gene is special since there are two homologs, so there are more predictions than in other cases.

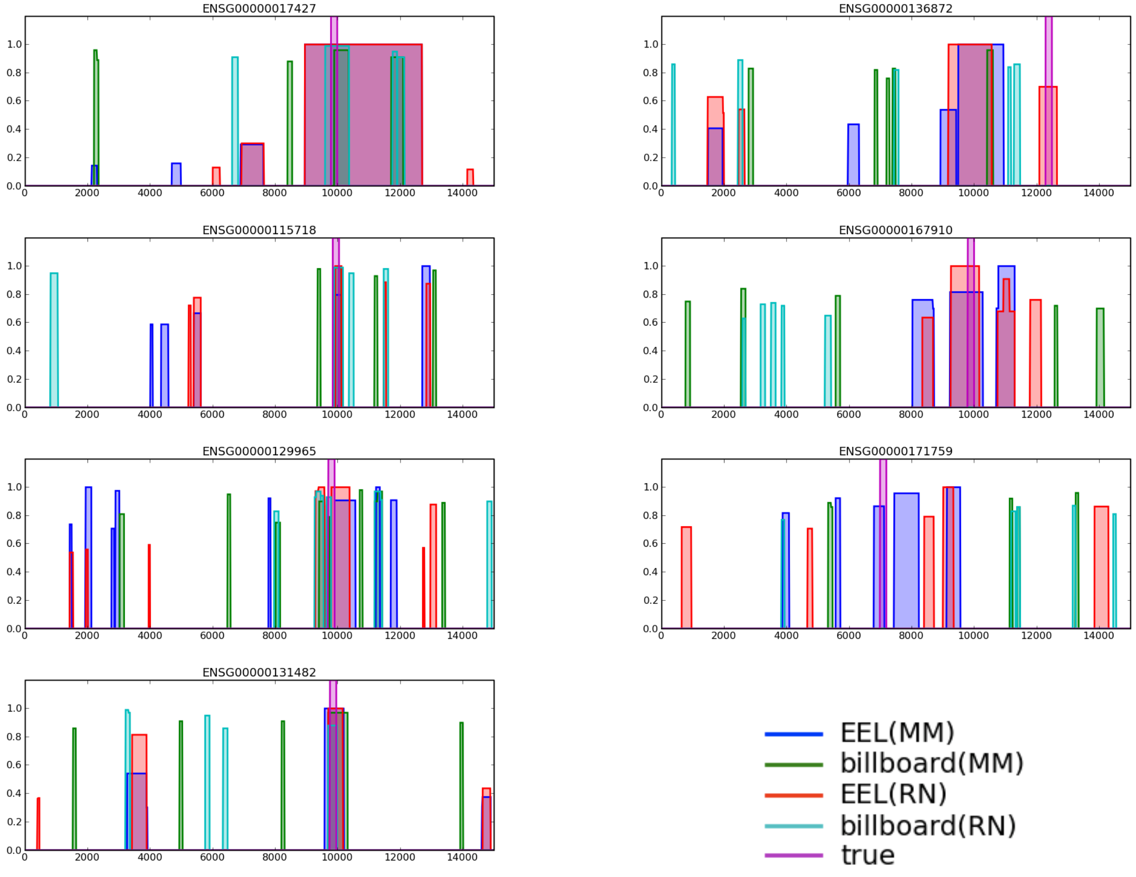

Figure 2: Top predictions of the EEL and Billboard methods on the liver dataset.

To summarize the results of liver-specific CRM predictions, we reproduce here an extended version of Table 1 from the text. For each gene name in column 1, one row shows quality of CRM prediction for one homolog (described in column 2). Quality of the most significant prediction which overlaps the experimentally verified CRM is presented by three attributes: rarity (see Section on Assessing the rarity of CRMs), position in ranking w.r.t. rarity (for *ex aequo* positions we show the range), and overlap.

Human gene	homolog species	rarity	ranking	overlap
ALDOB	Mouse	0.95	106-111	0.28
	Rat	0.90	83	0.97
IGF1	Mouse	0.04	1-2	0.29
	Rat	0.01	1	0.42
PAH	Mouse	0.98	98-101	0.64
	Rat	0.94	79-89	0.06
PROC	Mouse	0.01	1-2	0.64
	Rat	0.01	1-2	0.64
CYP7A1	Mouse	0.88	56-58	0.38
	Rat	0.84	62-63	0.83
G6PC	Mouse	0.03	1	0.49
	Rat	0.12	4	0.82
INS	Mouse	0.48	23	0.29
	Mouse	0.16	4	0.29
	Rat	0.21	4	0.29
	Rat	0.07	3-5	0.53

Table 4: Summary of liver-specific CRM predictions

.

6 ROC curves for Muscle and Liver datasets

6.1 Muscle dataset

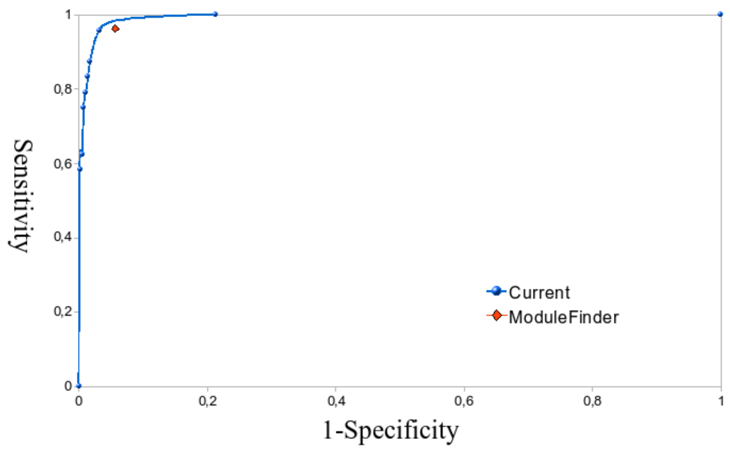

ROC curve showing the performance of our method on the training dataset (muscle). For comparison we included the result of the ModuleFinder method also trained on this data as published by Philippakis et al. (2005)

6.2 Liver dataset

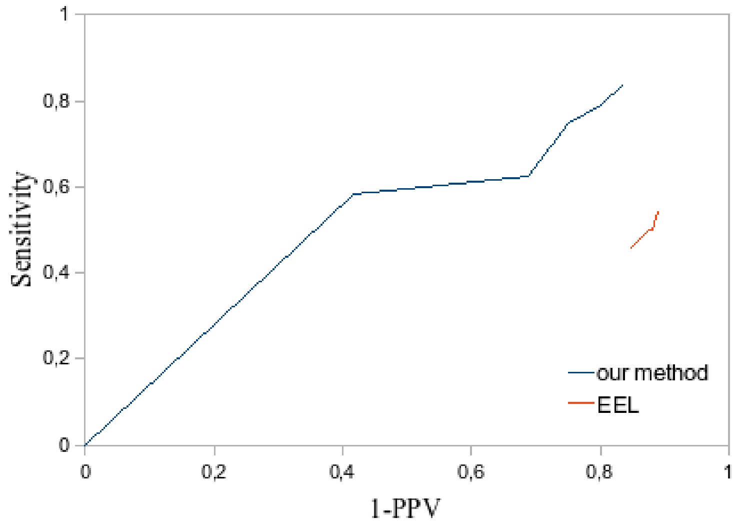

ROC curve showing the performance of our method on the liver dataset. For comparison we included the result of the EEL method using the same set of motifs.

7 Prediction summary for different Drosophila species (extended Table 2)

The table reports quality of the most significant prediction of each CRM in the gene *eve* in *Drosophila melanogaster* obtained by our method with each of other considered *Drosophila* species. The quality attributes are the same as in Table 4.

homolog	*Drosophila erecta*			*Drosophila ananassae*			*Drosophila pseudoobscura*			*Drosophila mojavensis*		
CRM	rarity	rank	overlap	rarity	rank	overlap	rarity	rank	overlap	rarity	rank	overlap
stripe3+7	0.22	8-11	0.54	0.06	6	0.62	0.01	1-3	0.54	0.04	7-8	0.54
stripe2	0.22	8-11	0.10	0.30	15-16	0.20	0.03	6-7	0.22	0.02	3-4	0.10
stripe4_6	0.12	4	0.21	0.10	8	0.88	0.02	4-5	0.53	0.03	5-6	0.65
stripe1	0.19	7	0.21	0.03	3-4	0.30	0.01	1-3	0.46	0.04	7-8	0.13
stripe5	0.23	12-13	0.27	0.30	15-16	0.26	0.61	29	0.21	0.34	23-24	0.09

8 Performance measures for Muscle and Drosophila dataset

Method	top ranked	found	SN	PPV
our	1	14	0.58	0.58
our	2	15	0.63	0.31
our	3	18	0.75	0.25
our	4	19	0.79	0.20
our	5	20	0.83	0.17
our	6	21	0.88	0.15
our	10	23	0.96	0.1
eel	1	1	0.04	0.04
eel	2	2	0.25	0.13
eel	3	10	0.42	0.14
eel	4	11	0.46	0.11
eel	5	12	0.50	0.1
eel	6	13	0.54	0.09
eel	9	14	0.58	0.06

Table 5: Performance measures for Muscle dataset

Method	Top ranked	found	SN	PPV
our	5	2	0.4	0.4
our	6	3	0.6	0.5
our	8	4	0.8	0.5
eel	3	1	0.2	0.33
eel	10	2	0.4	0.2
eel (specific)	5	5	1.0	0.8

Table 6: Performance measures for Drosophila dataset

9 Computational complexity

The algorithm of the billbard method might be decomposed into several stages. We will briefly outline their asymptotic complexity for a single analysis of two sequences of length N below:

1) **Reading input sequences** This step is linear with respect to the total length of sequences. We have a constant number of sequences of length N, so the complexity of this step is $\mathcal{O}(N)$.

2) **Annotating the sequences with motif occurences** We need to scan all sequences with all motifs from the database. If we assume that the length of motifs is bounded by a constant (in our case it is always $< 30bp$ with the median length ~ 10), the number of steps is linear with respect to both sequence length N and number of motifs M givinng time complexity of $\mathcal{O}(N \cdot M)$.

3) **Building the raw score matrix (V)** We are using a simple dynamic programming algorithm (outlined in Methods section), which computes simple set operations on every sequence window of a fixed size. The number of windows is N/J for each sequence, so we need to calculate $(\frac{N}{J})^2$ entries in the matrix. Each computation requires at most M basic operations, so the time complexity is $\mathcal{O}((\frac{N}{J})^2 \cdot M)$

4) **Calculating the rarity score** To calculate rarity, we repeat step 3) for a fixed number of random sequences ($\mathcal{O}((\frac{N}{J})^2 \cdot M)$) and then for each of the N/J windows, we calculate its maximum score value with the ranking of the randomized scores. This does not increase the asymptotic complexity, but it should be noted that the hidden constant is quite large (we use 100 random sequences).

In summary, the total time complexity of the algorithm is $\mathcal{O}((\frac{N}{J})^2 \cdot M)$, and if we consider the number of motifs in the database as constant, we obtain the complexity of $\mathcal{O}((\frac{N}{J})^2$. It should be noted that this cost includes a substantial multiplicative constant coming from the motif database size and the number of random sequences.

References

[1] Sven Rahmann, Tobias Müller, and Martin Vingron. On the power of profiles for transcription factor binding site detection. *Stat Appl Genet Mol Biol*, 2:Article7, 2003.

[2] Wyeth W Wasserman and Albin Sandelin. Applied bioinformatics for the identification of regulatory elements. *Nat Rev Genet*, 5(4):276–287, Apr 2004.
